# Designing an information technology system in public health: observations from India

**DOI:** 10.1186/1753-6561-6-S5-O19

**Published:** 2012-09-28

**Authors:** Sundararaman Thiagarajan, Pankaj Gupta, Amit Mishra, Itisha Vasisht, Alia Kauser, Dilip Singh Mairembam

**Affiliations:** 1 National Health System Resource Centre, Delhi, India; 2 Taurus Glocal Consulting, Delhi, India

## Introduction

The use of information technology in healthcare has seen varying success across states, due to differing institutional capacities. Despite isolated successes and partial gains, public health information technology systems in the country are unable to support integrated decision making at either district or state levels. An emphasis on technological advancements without significant attention to systems design has limited the potential gains from applications of information technology. This paper is an effort to describe the level of public health information technology systems development over the eleventh plan period and examine the architectural constraints that constitute a common problem across systems.

## Methods

All information technology systems serving any public health system need were listed. These were then categorized into types based on objectives and operational design features: - patient based reporting, aggregated reporting, disease reporting and family health survey based reporting. From this list, a representative set of eight major public health information technology systems were selected for the study. These were: national Health Management Information Systems (HMIS), mother and child tracking system at the national level and in Gujarat state, the information technology systems that support malaria, AIDS control and integrated disease surveillance programme and the state Health Management Information Systems in the states of Odisha and Tamil Nadu.

Functional efficacy was determined with the help of semi-structured functionality assessment tool which included parameters on user friendliness, data input-output, quality management functions, availability of analytical tools, flexibility of system and change management. Implementation and systemic aspects were then defined through discussion held with respective stakeholders.

## Results

Despite the influx of information technology in health, most of the systems have not achieved their objectives. Public health information technology systems have faced similar challenges of design and development. These challenges characterise a cyclical process of development that begins with high expectations followed by achievement of modest functionality, then increasing problems especially overload and finally a collapse to be followed by the next cycle of development.

Most systems required substantial changes after release, as programme managers were not skilled enough to articulate their requirements to software professionals. Data entry options lacked flexibility to adjust to different institutional capacities. This restricted data entry in the system. Data output options were also not flexible for different levels of users. Systems designs did not lend themselves to constantly changing programme reporting needs. Limited analysis functions were made available in seven of the eight systems examined.

We observed that all systems used their own data and reporting standards, which led to fragmentation and duplication. Data could not be shared between the systems, leading to a double burden of reporting for providers and loss of integration for the managers. Data security and privacy standards were not defined adequately across systems. Almost all information technology systems suffered from adoption issues due to poor capacity-building and change management.

## Discussion

The study revealed that public health information technology systems are working as reporting tools rather than programme management information systems. Various technical, organizational and capacity-building issues have been identified. System designs should prioritize the requirements of support to mid level programme managers and local users in their management activities other than reporting. This would bring substantial improvements in quality of data. All systems need to agree to some data and interoperability standards so that integration process can be initiated. Additionally focused capacity-building strategies are required to address adoption challenges. In this regard, a nationally acceptable electronic architecture based on standards of interoperability is required to be placed to help integration and standard application development in public health.

## Competing interests

Authors declare that they have no conflict of interest.

## Funding statement

This study was funded by the National Health Systems Resource Centre, New Delhi.

